# Integrating COVID-19 Vaccination in Primary Care Service Delivery: Insights From Implementation Research in the Philippines

**DOI:** 10.9745/GHSP-D-23-00202

**Published:** 2024-02-20

**Authors:** Juan Bernardo Lava, Vergil de Claro, Maria Socorro Quiñon, Rodney Labis, Wendel Marcelo, Miguel Angelo Lucero, Ophelia Mendoza, Laurentiu Stan

**Affiliations:** a RTI International Philippines, Pasig City, Philippines.; b Provincial Health Office, Province of Iloilo, Iloilo City, Philippines.

## Abstract

**Background:**

In 2019, the Philippines enacted a universal health coverage law that aimed to establish an integrated health system centered around robust primary care as a core strategy of its health system reform agenda. Although the COVID-19 pandemic disrupted initial progress in the reform process, it also presented an opportunity to pilot interventions to demonstrate integration in various ways.

**Methods:**

We conducted a participatory implementation research study to integrate selected public health interventions into the implementation of the primary care benefit package funded by public health insurance. The study was conducted from October 2022 to April 2023 in the Province of Iloilo, Philippines. Entry points within the primary care service delivery process were identified, and interventions related to COVID-19 vaccination and family planning were implemented and monitored. We used the RE-AIM (reach, effectiveness, adoption, implementation, and maintenance) framework to organize the results and present the analysis.

**Results:**

The intervention showed substantial improvement across the 5 dimensions of the RE-AIM framework, including enhanced access to health care services, as indicated by improvements in primary care patient registration, family planning services, and COVID-19 vaccination processes, as well as an increase in registrations and first patient encounters. These improvements corresponded to sustained primary care facility participation throughout the study period. Additionally, emergent factors that either impeded or facilitated the integration process were identified, providing insights for effectively integrating COVID-19 vaccination within the primary care health system.

**Conclusion:**

Our study provides evidence of the feasibility of integrating public health interventions into primary care settings. It highlights the potential of using existing primary care service delivery and financing mechanisms as entry points for integration. However, further iteration of the model is required to identify specific conditions for success that can be applied in other contexts and settings.

## Abstract (precis)

The authors provide evidence of the feasibility of integrating public health interventions into primary care settings and highlight the potential of using existing primary care service delivery and financing mechanisms as entry points for integration.

## Teaser

**Key Findings:**

- Existing primary care service delivery and financing mechanisms offer a practical and viable approach for integrating public health interventions, such as vaccination, within primary care settings.
- The successful implementation of the integration process requires substantial government support at various levels, active community engagement, and a multisectoral approach to regulations and strategies.

**Key Implication:**

- Evidence from this study can serve as a valuable resource for policymakers and implementers, enabling them to effectively design processes and allocate resources necessary for implementing an integrated primary health care system.

Plain language article summary available.

## INTRODUCTION

For over 4 decades, primary health care (PHC) has been recognized as a key strategy for achieving “health for all.”^1^^,^^2^ However, its implementation has faced challenges in translating the ambitious goals into tangible actions and results. There have been debates regarding the practicality of adopting a selective PHC approach over a comprehensive approach, of which the former was reinforced by targeted programs and interventions in the Millennium Development Goals.^3^ In the Astana meeting, the global community renewed its commitment to establishing a robust and comprehensive health system based on sustainable PHC and achieving universal health coverage (UHC).^4^ Countries agree that a PHC approach that prioritizes prevention and promotion, guarantees equal access to essential interventions, and reduces out-of-pocket spending on medication is the most efficient and equitable way to progress toward UHC.^5^

The global COVID-19 pandemic not only underscored the crucial role of primary care in effectively delivering essential and routine health services in response to emergency situations^6^^–^^8^ but also highlighted the potential synergies of a well-integrated PHC system.^9^ Earlier studies hinted that the lack of a cohesive health system within countries undermines their ability to withstand pandemics, such as COVID-19.^10^ This was observed in both high- and low-income countries. Despite scoring higher on the Global Health Security Index, some countries fared worse during the COVID-19 pandemic in terms of case numbers and fatalities compared to some countries in Asia and Africa,^11^ which scholars partly attribute the comparatively better performance to more effective integrated health systems, particularly at the PHC level.^12^^–^^13^ Yet even before the COVID-19 outbreak, several studies had already advocated for prioritizing integrated health systems over fragmented ones. This approach facilitates the seamless integration of essential building blocks, including global security, UHC, and PHC, within countries.^14^ This was the case in the Philippines, where PHC was considered a core reform and a bedrock of its UHC agenda. Its Universal Health Care Act aimed to implement an integrated and comprehensive set of health services, including promotive, preventive, rehabilitative, and palliative, to safeguard its citizens against financial risk as a national strategy for achieving UHC.^15^

In this article, we describe the design process, results, and lessons from implementing an integrated PHC model in the Philippines. Our findings offer valuable perspectives on essential factors to consider when incorporating vaccination services into existing primary care service delivery systems. Current evidence suggests that the integration of vaccination services within primary health care settings enhances their efficiency, accessibility, and equity, leading to improved health outcomes. Our findings serve as a practical guide for implementers and policymakers, furnishing them with insights and a roadmap for effectively merging public health interventions and primary care services.

## AN OPPORTUNITY TO MODEL PHC INTEGRATION

The COVID-19 pandemic disrupted the initial rollout of UHC reforms in the Philippines, but it also provided an opportunity to assess how integrated delivery of individual and population-based health services, coordination of care across service provider networks, and financing mechanisms for primary care were necessary health system responses to the health crisis.^16^^,^^17^ The Philippines operates in a decentralized system in which local government units (LGUs), which are provinces, cities, and municipalities, have governing and fiscal autonomy. It should be noted that delivery and financing of public health and primary care services in the country still operate independently. Vaccination activities are done separately from primary care consultations, with distinct funding sources. The national government typically funds vaccination, and LGUs finance primary care and are reimbursed by public health insurance. The national government often deploys vaccinators to assist LGUs with vaccination activities, leading to competing priorities depending on the immediate government directives, such as the focus on COVID-19 vaccination during the pandemic.

**FIGURE 1:**
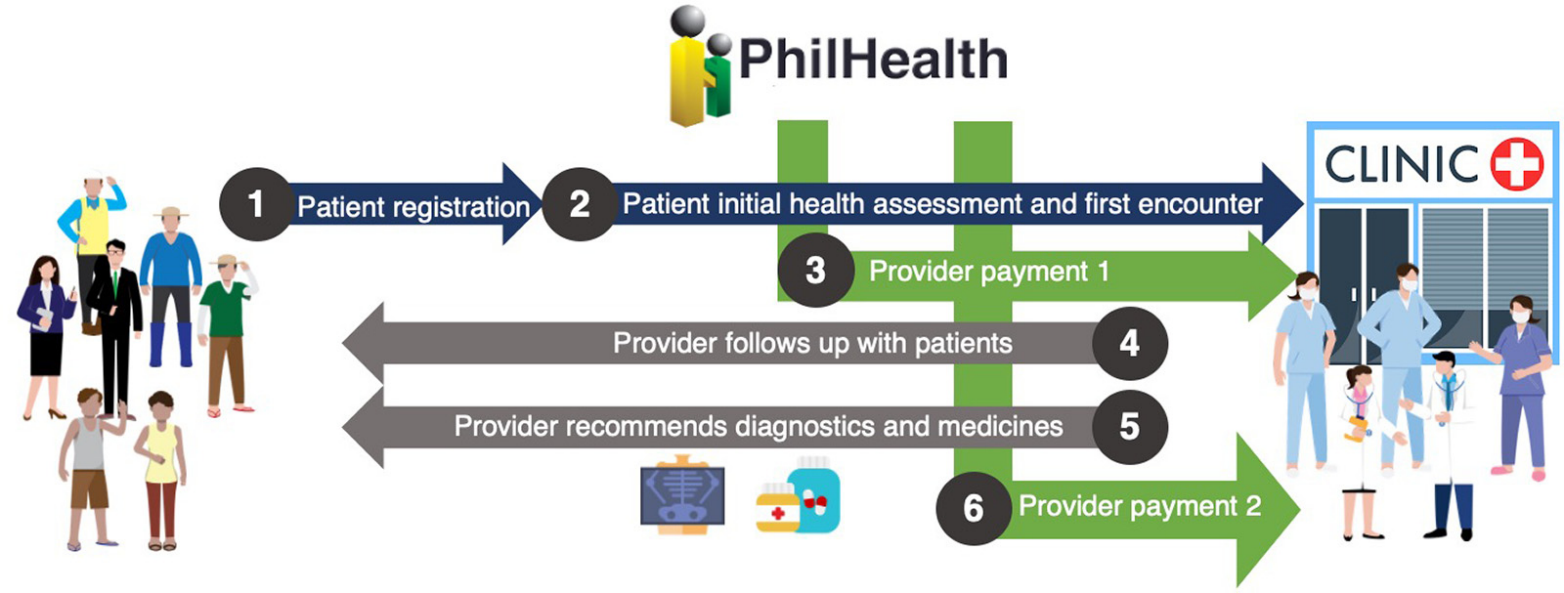
PhilHealth’s Primary Care Benefit Package Services and Payment Mechanisms Abbreviation: PhilHealth, Philippine Health Insurance Corporation.

The Philippine Health Insurance Corporation (PhilHealth), which is the public health insurance agency, rolled out a primary care benefit package known as Konsulta that reimburses the LGU for a set of primary care services it provides (Box, Figure 1).^18^ This package provided the avenue for designing a service delivery model in which public health and primary care services were integrated into service delivery and funding. In terms of service delivery, the package facilitated the seamless capture of patient information, efficient service delivery, and effective referral processes, promoting a team-based approach to care. In terms of funding, the package offered sustainable financing for primary care services that would have otherwise been an annual expense of the LGU.

BOX Philippine Health Insurance Corporation Primary Care Benefit Package

Philippine Health Insurance Corporation’s (PhilHealth) primary care benefit package, Konsulta, is an expanded set of preventive and promotive health care services and is made available to all Filipinos registered to accredited public and private primary care providers (Figure 1).

**Package Inclusion:**

- Initial and follow-up consultation with a primary care provider.
- Targeted health risk screening and assessment for asthma, acute gastroenteritis, upper respiratory tract infection, urinary tract infection, TB, breast and cervical cancer, diabetes, hypertension, and dyslipidemia.
- Thirteen diagnostics (e.g., blood and serum chemistry, imaging, glucose test, and cardiovascular tests) and 21 essential drugs (e.g., rehydration salt, antibiotic, antithrombotic, antihistamine, antihypertensive, antidiabetic, and antidyslipidemic).

**Payment Mechanism:** Providers in primary care facilities are paid about US$10–13 annually per capita (with differentiated pricing for public and private providers) in 2 tranches:

1. Payment 1 (40% of total payment) is a fair fixed cost paid after conducting initial health assessment and first patient encounter.
2. Payment 2 (60% of total payment) is performance based after completing the required services for consults, diagnostic tests, and prescription of antibiotics and noncommunicable disease medications based on patient health profile data.

**Provider Participation:** Primary care facilities that have the capacity and are willing to provide the services under the Konsulta package are required to apply for accreditation to participate. The accreditation standards include requirements for service capability for preventive/screening, laboratory and radiologic services, infrastructure, infection control, equipment and supplies, human resources, and meeting the reporting and recording requirements using a PhilHealth-certified health information system.

The Konsulta primary care benefit package provided the avenue for designing an integrated service delivery model for service delivery and financing.

The ReachHealth Project, funded by the U.S. Agency for International Development (USAID) and implemented by RTI International, provided technical support to the Provincial Government of Iloilo to develop and implement an integrated health service delivery model consisting of COVID-19 vaccination and family planning services—which are both priority programs of the province and technical assistance interests of USAID—alongside the PhilHealth primary care benefit package.

## METHODS

This study uses a participatory implementation research approach. Implementation research plays a vital role in enhancing our comprehension of the obstacles encountered in real-world scenarios, as it expands and deepens our understanding of these real-world factors and their influence on implementation and is immensely valuable in highlighting the complex relationship between theory and practice.^19^

### Study Setting

**FIGURE 2:**
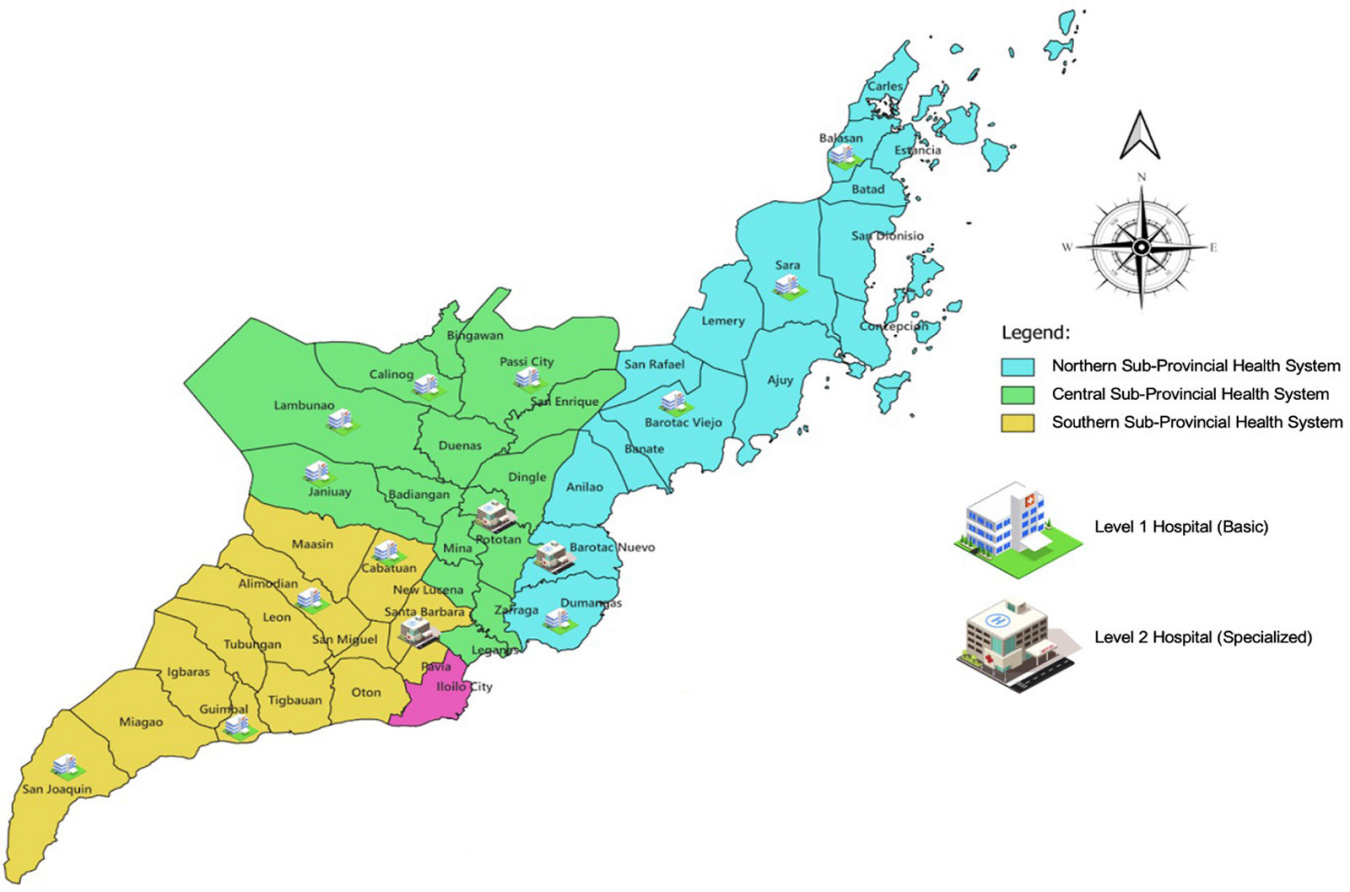
Map of Province of Iloilo, Philippines Showing Public Health Facilities and Clusters of Subprovincial Health Systems

The study was conducted within the Province of Iloilo, located in the Western Visayas region of the Philippines. Positioned at the heart of the archipelago, Iloilo has a population of approximately 2.1 million and is composed 1 city and 42 municipalities. The LGUs with their respective public health facilities were clustered into subprovincial health systems (Figure 2).

### Intervention Description

**FIGURE 3:**
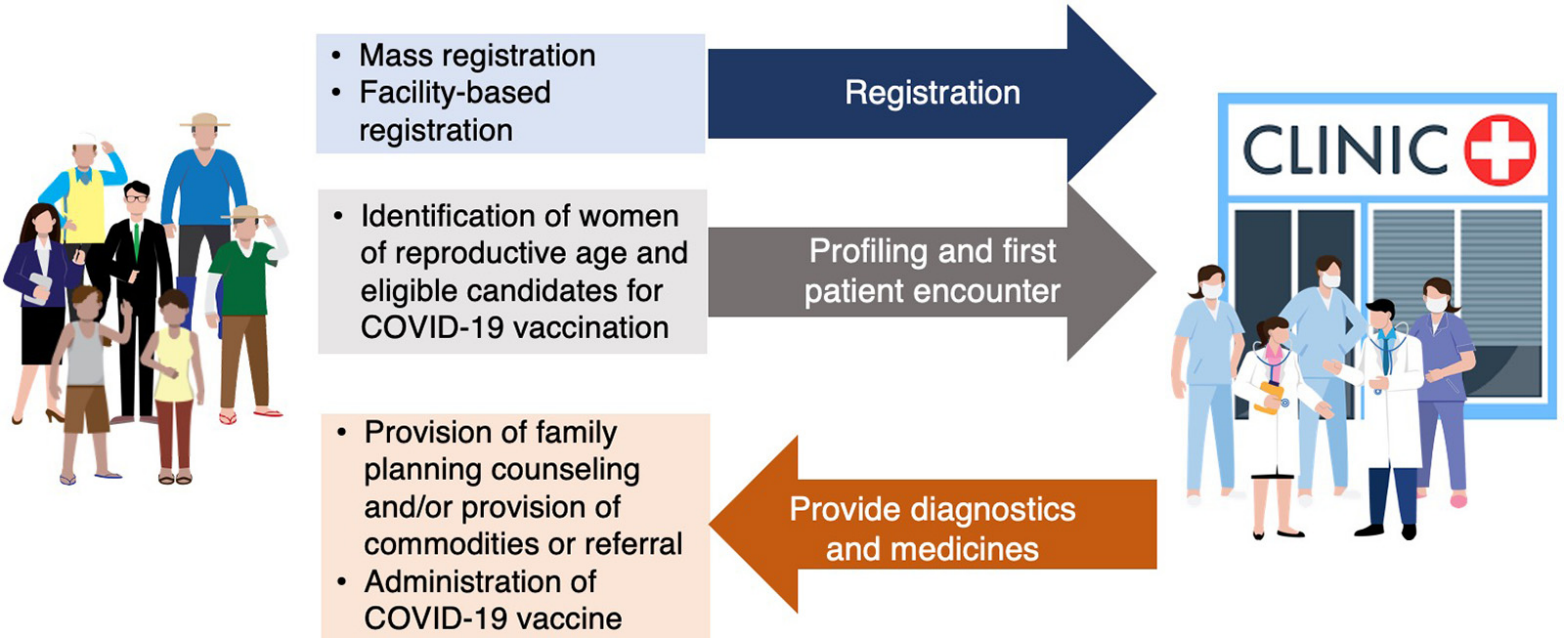
Public Health and Primary Care Integration Model in Province of Iloilo With Interventions Shown Across the Service Delivery Process

We identified 3 entry points for integrating public health interventions within the service delivery process of the primary care benefit package: (1) streamlining patient registration through mass registration, which targets a significant portion of the population through organized events or campaigns (Supplement), or through facility-based registration, which involves the regular process of registering individuals at the health care facility and enables a more personalized and individualized approach; (2) identifying women of reproductive age and eligible candidates for COVID-19 vaccination during the profiling and first patient encounter stage; and (3) providing family planning counseling and services (onsite or through referral to a higher-level facility) and administering COVID-19 vaccines to those who want to receive these services (Figure 3).

These interventions were implemented from October 2022 to April 2023 in the following 3 phases.

#### Phase 1: Planning

The planning and codesign process was led by the provincial health office, which coordinated the involvement of LGUs in the province. Before the intervention was implemented, baseline quantitative data were collected and used to project certain targets. In the first 3 months, 20 LGUs participated in the integrated approach, followed by an additional 16 LGUs in the next quarter, for a total of 36 LGUs. To support the implementation of the intervention, 36 medical consultants were recruited to work in collaboration with the health staff at each primary care facility and function as integral members of the team under the direct supervision of the municipal health office. All medical consultants were licensed physicians who received training on the approach and interventions before the implementation. They were tasked with overseeing Konsulta patient registration, which involved gathering comprehensive medical histories and performing physical examinations; encoding clinical data directly into the primary care database or using paper-based forms; screening individuals for COVID-19 vaccination status and family planning needs; providing counseling on vaccination and family planning; administering vaccines when necessary; and managing any adverse events following immunization. The ReachHealth Project funded the employment of the medical consultants.

#### Phase 2: Implementation

Each LGU was assigned a medical consultant who worked closely with the health managers to coordinate registration activities and implement the interventions alongside the delivery of primary care services. The planning and execution involved all primary care facility personnel (managers, nurses, and community health workers), representatives from the regional Department of Health and PhilHealth offices, and community leaders.

#### Phase 3: Monitoring and Evaluation

Quantitative data were collected from each primary care facility database and consisted of registration and service outputs for COVID-19 vaccination, family planning, and the primary care services in the Konsulta package. The consultants submitted monthly reports to monitor progress, challenges, and adaptive practices. We conducted 2 focus group discussions (FGDs) with medical consultants; representatives from the LGU, provincial health office, regional Department of Health, and PhilHealth offices; and ReachHealth Project personnel. The FGDs were used to collect qualitative data on facilitators and barriers to implementation, adaptive measures taken, and alternative solutions to explore. A survey was also introduced in the last week of implementation to collect information on vaccine hesitancy.

We used the RE-AIM (reach, effectiveness, adoption, implementation, and maintenance) framework to evaluate the impact of our intervention in terms of its reach, effectiveness, adoption, implementation fidelity, and maintenance in care (i.e., sustainability of the program).^20^ By using RE-AIM, we efficiently planned, evaluated, and assessed the outputs of the intervention, including its effectiveness and sustainability.

### Data Collection and Analysis

PhilHealth registers and forms were adapted in this study. Patient information was collected from all the primary care facilities in the study sites and encoded in health information systems certified by PhilHealth. The certification guarantees the compliance of the health information system with the standards and specifications on system requirements and data integrity, ensures adherence to data privacy and confidentiality policies, and provides appropriate safeguards (e.g., encryption, storage, and backup). Only anonymized and aggregated data were used for analysis. The evaluation of outcomes was done by comparing projected targets and post-implementation results (Table 1).

**TABLE 1.** Summary of Project Performance, Province of Iloilo, Philippines, as of April 30, 2023

**RE-AIM Dimensions/ Indicators**	**Projected,** **No. (%)**	**Post-Intervention Performance, No. (%)**	**Means of Verification**	**Remarks**
Reach				
Proportion of population that was registered to a primary care provider^a^	420,000 (≈20.0)	405,826 (19.3)	Facility-level data and program reports	The cumulative total population registered with a primary care provider includes individuals registered before the study. However, a substantial portion of the overall result can be attributed to the interventions implemented during the study.
Effectiveness				
Number of individuals that had FPE (including health profiling)	72,000 (100.0)^a^	110,795 (153.9)	Facility-level data; program reports	Our observations indicate that the rotating barangay-based model, which involved conducting scaled-down mass registration activities in each barangay (village) without the need for additional personnel, had a greater impact on the increase in first-patient encounters compared to both continuous facility-based model and mass registration approaches.
Number of WRA given FP services	3,240 (100.0)^b^	10,369 (320.0)	Facility-level data; program reports	The total number of WRA with contraceptive methods initiated was not accurately tracked. However, we were able to document 1,099 WRA as new or other acceptors, and the remaining only received FP counseling services.
Number of individuals given COVID-19 vaccination	33,957 (100.0)^c^	15,628 (46.0)	Facility-level data; program reports	The primary COVID-19 vaccine series coverage was already high at the start of the study. However, the lower-than-expected results can be attributed to challenges in closely monitoring adaptive measures for vaccination and vaccine supply shortages during the study period.
Adoption				
Number of primary care facilities who participated in the intervention	43 (100.0)	36 (81.4)	Program reports	Only 36 of all 43 public primary care facilities participated in the study. Among the 36 facilities, 27 were accredited by PhilHealth by the end of the intervention.
Implementation				
Fidelity (adherence to steps and intervention protocol)	Consistency between recommended and implemented processes in the intervention protocol	See remarks	Minutes of mid-implementation review and pause-and-reflect sessions; focus group discussion transcripts	Modifications in the registration activities were made. Initially, the registration format consisted of 2 designs: (1) facility-based registration and profiling setup and (2) a mass registration activity held once per local government unit with additional workforce support. Adopted a patient-centric approach through the rotating barangay-based model. Also, a vaccine hesitancy survey was later introduced to gather additional information on the topic.
Maintenance				
Number of primary care facilities continuing to implement the intervention after the study period	36 (100.0)	36 (100.0)	Observation checklist; post-implementation review; facility-level data	To date, the facilities are actively implementing the intervention, and the ReachHealth project continues to provide the necessary technical assistance to ensure sustained implementation in these sites. Furthermore, a select number of consultants were hired directly by the primary care facilities in the months after the engagement as full-time staff and continue to implement the model.
Increase in the amount of health insurance reimbursements	No target indicated; baseline was <PhP4,000 or US$80	PhP553,915 or US$11,078	Observation checklist; post-implementation review; facility-level data	At the beginning of the activity, the participating health facilities generated less than PhP4,000 in health insurance reimbursements. The significant increase at post-intervention assessment was considered a critical driver for sustained financing of the intervention.

Abbreviations: FPE, first patient encounter; FP, family planning; PhilHealth, Philippine Health Insurance Corporation; PhP, Philippine peso; WRA, women of reproductive age.

^a^ Based on a 30–35 daily average count of FPE considering the available medical consultants and number of days allocated to conduct the FPE within the intervention period.

^b^ Based on the proportion of WRA among the target population estimated using the family planning estimation tool; potential reach was adjusted for an anticipated higher attendance, particularly among stay-at-home mothers, given the type of outreach activities.

^c^ Based on the estimated reach of 10% of eligible individuals who had not received COVID-19 vaccination among the target population.

### Ethical Approval

Because our research aimed to improve health service delivery without using personal data, obtaining ethical clearance was deemed unnecessary. LGUs voluntarily participated, were authorized by their respective local legislative councils, and remained engaged throughout. Medical consultants signed confidentiality agreements, and all data were securely stored in an institutional cloud server.

## RESULTS

Within the 6-month period (October 2022 to April 2023), a total of 84% (n=36) of the LGUs in the Province of Iloilo actively participated in the implementation research. Among the 36 primary care facilities, 27 facilities (75.0%) received accreditation from PhilHealth to provide Konsulta services to their constituents. This represented an increase of 80% in the number of accredited facilities from the initial 15 accredited facilities at the start of the study period.

Table 1 presents a summary of the performance of the integrated approach. The registration activities contributed to capturing 405,826 (19.8%) of the target population, of which 100,795 (27.3%) underwent health profiling and received their initial patient consultation. Of these profiled individuals, 15,628 (14.1%) received COVID-19 vaccination, and 10,369 (9.4%) received family planning counseling and services. The amount of health insurance reimbursements from PhilHealth increased from 4,000 Philippine pesos (PhP) to PhP553,915. All 36 sites completed the implementation of the intervention during the entire study period.

### COVID-19 Vaccination Performance

Before the start of the intervention, COVID-19 vaccinations in Iloilo had already reached a significant primary series coverage rate, with 85.6% for individuals aged 18–59 years and 75.3% for individuals aged 60 years and older (although still falling short of the national target of 90.0%). However, when it came to the first booster coverage, the rates remained below the target of 50%, with only 23.0% coverage for people aged 18–59 years and 21.6% for individuals aged 60 years and older.

The integrated service delivery contributed to an additional 15,628 vaccinations (46.0%) of the set target. Based on vaccine type, the majority of doses were given as first boosters, accounting for 8,472 (54.2%) of total doses given. The primary series constituted 4,512 (28.9%), and the second booster represented 2,644 (16.9%) of the total doses administered. Analyzing the data further by age group, the highest proportion of doses were given to individuals aged 18–59 years, comprising 10,233 (65.5%) of all doses administered. Children aged 5–11 years received 2,645 (16.9%) of the total doses, followed by 1,768 (11.3%) doses to individuals aged 60 years and older. Children aged 12–17 years accounted for the smallest number of doses at 982 (6.3%) of all doses administered.

The integrated service delivery contributed to an additional 46.0% vaccinations of the set target.

**FIGURE 4:**
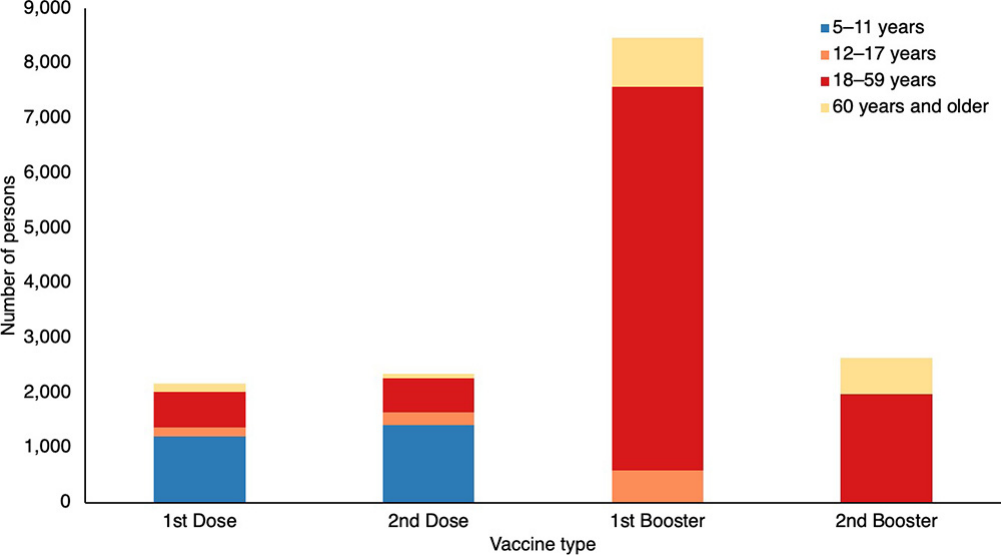
Distribution of COVID-19 Vaccine Doses Across Age Groups and Vaccine Types, Province of Iloilo, Philippines

Among those who received the primary series vaccine, children aged 5–11 years received the majority of doses, accounting for up to 2,629 (58.3%) of all first and second doses given. In contrast, individuals aged 60 years and older received the lowest number of doses at 218 (4.8%) of all first and second doses given (Figure 4).

**FIGURE 5:**
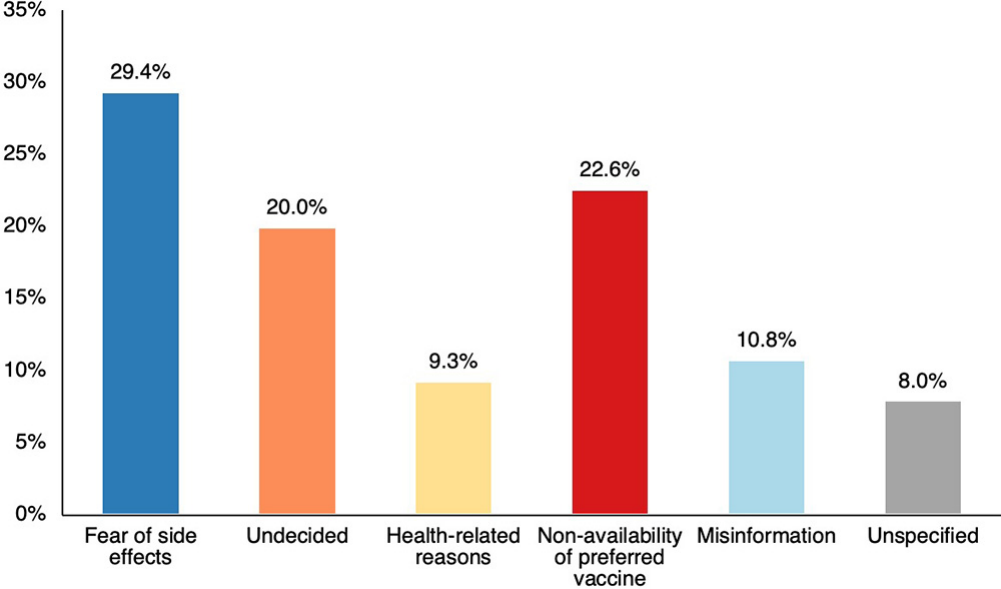
Reasons for Vaccine Hesitancy, Survey Results, Province of Iloilo, Philippines^a^ ^a^ Survey conducted in March 2023.

Findings from the vaccine hesitancy survey showed that of 2,427 respondents, 713 people (29.4%) expressed their primary concern as fear of experiencing side effects, and 548 people (22.6%) cited the nonavailability of their preferred vaccine as the reason for refusal (Figure 5). Challenges associated with expiration and maintaining cold chain storage during community-based activities further contributed to shaping this perception. Additionally, 20.0% of respondents were undecided, indicating that family decision-makers’ perspectives, especially for children and seniors, were necessary to consider in determining whether to proceed with vaccination.

### Barriers and Facilitators for Integration

Based on data from the FGDs with implementers, we synthesized the emergent factors that either impeded or facilitated the integration process, organized according to the RE-AIM dimensions (Table 2). The FGD participants identified several elements that were crucial for the successful integration of COVID-19 vaccination, including closely collaborating with community actors supporting community mobilization activities, conducting information campaigns, allocating additional human resources for vaccination efforts, and ensuring the availability of sufficient vaccine supply and efficient logistics. They also reported barriers such as insufficient backing from local leaders, resource constraints, negative community perception toward the program, issues with the health information system, and inadequate national-level policy support and technical guidance.

**TABLE 2.** Facilitators and Barriers to Implementing an Integrated Primary Care Service, Province of Iloilo, Philippines

**RE-AIM Dimensions**	**Facilitators**	**Barriers**
Reach	Endorsement from the municipal health officer and mayor Availability of human resources for health Positive reception of the Konsulta package by local health teams Close proximity of the primary care services to households	Lack of support from local chief executive Insufficient human resources Inadequate financial support for conducting mass registration activities Considerable distance from the site of registration and profiling
Effectiveness	Availability of vaccines and family planning commodities Involvement of the family decision-maker Presence of physician for patient persuasion, in contrast to other health care workers	Misinformation by organized local groups Absence of vaccines and family planning commodities Timing of profiling activity (weekdays meant fewer working individuals and more senior citizens)
Adoption	High interest of municipal health officers Provision of incentives for municipalities committed to implementation (i.e., augment human resources) Initial funding for the hiring of medical consultants to provide technical support	Obtaining approval from local legislative body to participate in the intervention takes time Unfavorable perception of the current capitation amount of the primary care benefit package considered to be below the market cost
Implementation	Existence of efficient knowledge-sharing channels through multistakeholder group chats and frequent meetings Effective utilization of weekly reports and regular feedbacking of implementation challenges	Lengthy process in securing primary care facility accreditation from public health insurance Lack of resources to conduct mass registration in certain settings
Maintenance	Commitment of local chief executives and municipal health officers to generate reimbursements from implementing the intervention	Lack of support from local chief executives Inadequate national-level support to address broader issues, such as accreditation of electronic medical records and changes and agile policies to respond to diverse circumstances

Abbreviation: RE-AIM, reach, effectiveness, adoption, implementation, and maintenance.

## DISCUSSION

We aimed to document a PHC model that integrated COVID-19 vaccination within the primary care benefit package funded by the public health insurance system. The findings from this study demonstrate the promising potential for seamlessly integrating public health interventions within primary care settings, which serve as the first point of contact for individuals.^21^ Previous successful implementation of vaccination programs by primary care,^22^^–^^24^ along with recent evidence highlighting their ability to bridge the service delivery gap for COVID-19 vaccines,^17^^,^^25^^,^^26^ further supports this potential.

The integration model we piloted has considerable potential as a proof of concept for the various aspects of delivering and financing integrated health services. First, it showcases the seamless integration of diverse public health services into a disease-agnostic primary care package.^27^ Second, the intervention shows substantial progress across the 5 dimensions of the RE-AIM framework, particularly in enhancing access to health care as indicated by improvements in patient registration and the first patient encounter and in access to family planning corresponding to sustained primary care facility participation in all LGUs. Monitoring family planning services within primary care environments serves as an effective surrogate measure for primary care access. Third, the intervention mirrors the accessibility, acceptability, and affordability of services for a wide spectrum of the population and carries substantial public health implications.^28^^,^^29^ Challenges in COVID-19 vaccination coverage were also noted related to vaccine supply and logistics issues and persisting vaccine hesitancy. Finally, the integration model’s adoption and maintenance among pilot sites were also substantial, particularly in the amount of health insurance reimbursements, which indicates promising prospects for its sustainability. We have demonstrated that by understanding the existing financing mechanism for primary care and optimizing its workflow processes to provide integrated care, LGUs were able to enhance service efficiency and local resource allocation, thereby generating ample insurance reimbursements, which they can reinvest to support the expansion and long-term sustainability of the initiative. Overall, these results highlight the viability of consolidating different health services within a unified primary care framework, enabling efficient and coordinated care delivery. Therefore, our model also presents a compelling case for the inclusion of individual-based interventions with significant public health impact into the public health insurance package. This is important considering the plan to transition the current primary care package of PhilHealth into a comprehensive outpatient benefit coverage package that aims to integrate other essential health services into the package, making health care accessible and affordable to a larger population.

The model we piloted showcases the seamless integration of diverse public health services into a disease-agnostic primary care package.

We found that the PHC operational framework^30^ was a useful framing for organizing the experience and insights around the key components involved in implementing an integrated primary care system. First, our findings highlight the need for extensive governmental support at multiple levels to effectively implement integration. Among the identified facilitators are the endorsement from local chief executives and local health authorities and commitment to allocating resources to improve the health infrastructure, recruitment of additional health workforce, and provision of training to enhance the skills and competencies of health workers tailored to the specific needs of primary care settings. Additionally, the national government should prioritize its support toward enabling technologies to effectively capture and use data to facilitate evidence-based decision-making, provide technical guidance, and build the capacities of LGUs to design integrated model that is tailored to their context.

Second, COVID-19 has underscored the crucial role of community engagement and empowerment.^31^^,^^32^ We recognized this as a pivotal driver in the implementation of our integration model. Rather than imposing a predetermined implementation design from the outset, we actively sought inputs from various stakeholders at different stages of the process. The conduct of progress check-ins and the midproject review allowed us to capture the perspectives and insights of the relevant stakeholders at each point in time. For example, the rotating barangay-based model as an additional approach to registration that prioritizes patient-centered care and maximizes the available pool of resources from multiple sectors (e.g., community health workers, village personnel, and schools) was codeveloped with facility personnel and feedback from patients. Similarly, we found value in using a community-based approach to tackle vaccine hesitancy concerns and having local primary care practitioners play a pivotal role. Researchers have already noted that community-based practitioners are widely trusted as a source of health information, making primary care venues dependable platforms for communicating accurate information about vaccines and addressing similar public health challenges within their communities.^25^^,^^26^ A significant portion of our survey finds common reasons that contribute to vaccine hesitancy that can be effectively addressed through persuasive communication by trusted primary care practitioners, community health workers, community centers, and local institutions that can expedite the attainment of vaccination equity, even for those individuals who may present the greatest challenges in terms of accessibility.^25^

Third, we emphasize the importance of addressing the broader health determinants in establishing a comprehensive and integrated PHC system, which requires evidence-based policies and actions implemented in a multisectoral setting.^33^ There is a growing body of evidence in this area being demonstrated in a number of countries that have established task forces and coordinating structures with both vertical and horizontal links across multiple sectors.^34^^,^^35^ At the national level, the Philippines has made great strides in terms of establishing a policy framework that fosters a whole-of-system, whole-of-government, and whole-of-society approach in implementing its UHC reforms.^15^ In the Province of Iloilo, the planning, design, and implementation of our integrated approach highlights the value of multistakeholder cooperation among national and local governments, development organizations, community leaders, and the public, paving the way for timely and transparent access to information, resources, and mechanisms of action among stakeholders, an approach aligned with the guidance of the World Health Organization on integration.^36^

We emphasize the importance of addressing the broader health determinants in establishing a comprehensive and integrated PHC system, which requires evidence-based policies and actions implemented in a multisectoral setting.

In summary, the COVID-19 crisis has yielded valuable insights into the importance of integrating vaccination services within existing primary care service delivery and financing mechanisms in countries. It can be argued that the challenges arising from the pandemic have accelerated necessary and long-awaited changes in PHC systems. Therefore, it is essential to prioritize and expedite efforts to integrate vaccination services into primary care settings. The PHC operational framework serves as a valuable resource to guide country-level actions toward a PHC-oriented health system. This transformation will not only enhance the response to future health emergencies and outbreaks but also accelerate the achievement of UHC.

### Strengths and Limitations

This study adds to the existing global evidence on the value of integrated health care systems, specifically in low- and middle-income settings. It highlights the significance of a locally led, multisectoral, and contextually relevant approach to integration. The study’s strength lies in its participatory design and adaptive process during the intervention’s implementation. However, the alignment of the study’s results with the proposed actions outlined in the strategic and operational levers of the PHC operational framework has not been extensively examined. Moreover, a potential constraint on the replicability of the intervention arises from the initial funding of medical consultants by a donor, as it necessitates the LGU to make an initial investment with the anticipation of generating revenues that can be channeled back to finance it in the long term. Likewise, due to the study’s short duration, the impact of integration on individual-level health outcomes could not be measured. These aspects should be further explored in future studies.

## CONCLUSION

This study demonstrated the feasibility of integrating public health interventions into primary care settings. The results highlight the potential of leveraging existing primary care service delivery and financing mechanisms as effective entry points for integration. However, further iteration of the integration model is necessary to identify specific conditions for success applicable in other contexts and settings. Policymakers and implementers are encouraged to embed a learning system within intervention processes to foster continuous improvement, refine approaches, and explore strategies for integrated PHC.

## Supplementary Material

23-00202-Lava-Supplement.pdf

GHSP-D-23-00202-supplement3.pdf

GHSP-D-23-00202-supplement1.pdf

GHSP-D-23-00202-supplement2.pdf

## Notes

**Peer Reviewed**

**First published online:** November 10, 2023.
