# Supplementary material for: Integrating COVID-19 Vaccination in Primary Care Service Delivery: Insights From Implementation Research in the Philippines
Source: Glob Health Sci Pract. 2024 Feb 20;12(Suppl 1):e2300202. doi: 10.9745/GHSP-D-23-00202 (PMC10948126; doi:10.9745/GHSP-D-23-00202)
Supplement: 23-00202-Lava-Supplement.pdf [file 23-00202-Lava-Supplement.pdf]

Supplement to: Lava JB, Quinon MS, Stan L, et al. Integrating COVID-19 vaccination in primary care service delivery: insights from implementation research in the Philippines. *Glob Health Sci Pract*. 2023;11(Suppl 4):e2300202. https://doi.org/10.9745/GHSP-D-23-00202

SUPPLEMENT. Mass Registration Process

	Preparatory Activities	**Step 1** Registration Check	**Step 2** Data Capture	**Step 3** Patient Consultation and Referral	**Step 4** FP Counseling and/or COVID-19 Vaccination
**Tasks**	• Partner coordination and tasking<br>• Pre-targeting of registrants<br>• Logistics - venue, food, forms, equipment etc.	• Check patient name in PhilHealth database	• Collect patient personal information using the paper forms<br>• Encoding of data using PHC information system	• Perform health assessment and physical examination	• Conduct FP counseling and actual service provision and/or<br>• COVID-19 vaccination advocacy and actual jab
**Materials**	• Planning checklist	• List of target participants	• Registration forms	• Consultation sheet; FP/COVID-19 screening tools	• Advocacy materials
**Duration**	• 2 weeks before conduct of activity	• 5 to 10 minutes	• 10 to 15 minutes	• 15 to 30 minutes	• 30 to 90 minutes
**Responsible**	• Provincial Health Office and LGU concerned	• Regional PhilHealth and LGU	• Regional PhilHealth and LGU	• Medical consultant and primary care facility personnel	• Medical consultant and primary care facility personnel
