# Supplementary material for: Integrating COVID-19 Vaccination in Primary Care Service Delivery: Insights From Implementation Research in the Philippines
Source: Glob Health Sci Pract. 2024 Feb 20;12(Suppl 1):e2300202. doi: 10.9745/GHSP-D-23-00202 (PMC10948126; doi:10.9745/GHSP-D-23-00202)
Supplement: GHSP-D-23-00202-supplement3.pdf [file GHSP-D-23-00202-supplement3.pdf]

GHSP Lições da integração da vacinação contra a COVID-19 nos cuidados de saúde primários | Série de resumos de artigos

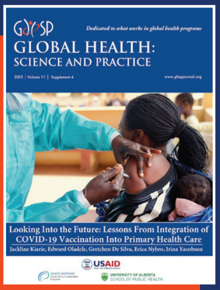

Integração da vacinação contra a COVID-19 na prestação de serviços de cuidados primários: conclusões do estudo de implementação nas Filipinas: resumo do artigo

Juan Bernardo Lava, Maria Socorro Quiñon, Rodney Labis, Wendell Marcelo, Miguel Angelo Lucero, Ophelia Mendoza, Laurentiu Stan, Vergil de Claro

De que trata este artigo? Os serviços de vacinação contra a COVID-19 e de planeamento familiar foram integrados no programa de prestação de cuidados de saúde primários existente nas Filipinas, que é financiado pelo sistema de saúde público.

Quais foram os resultados? O acesso aos serviços de saúde melhorou, o acesso ao planeamento familiar melhorou, o número de pacientes registados aumentou e o número de primeiros encontros de pacientes com profissionais de saúde aumentou. A integração das vacinas contra a COVID-19 no modelo de prestação de serviços resultou em 15.628 vacinações adicionais contra a COVID-19, 46% do objetivo. A maior parte destas doses foram primeiros reforços que envolveram setores marginalizados. O montante do reembolso do seguro de saúde da PhilHealth aumentou de 4.000 pesos filipinos (PhP) para 553.915 PhP em 6 meses.

O que significam estes resultados? Os resultados oferecem perspetivas valiosas sobre os fatores essenciais que os decisores políticos devem considerar ao incorporar os serviços de vacinação nos sistemas de cuidados primários existentes. Por exemplo, a utilização de programas de prestação de serviços de cuidados primários e de mecanismos de financiamento existentes constitui uma forma prática de integrar intervenções de saúde pública, como a vacinação, em contextos de cuidados primários.

Perspetivas dos autores

O projeto ReachHealth é implementado pelo Research Triangle Institute (RTI) International nas Filipinas e é financiado pela USAID.

"É possível argumentar que os desafios decorrentes da pandemia aceleraram as mudanças necessárias e há muito esperadas nos sistemas de cuidados de saúde. Por conseguinte, é essencial dar prioridade e acelerar os esforços para integrar os serviços de vacinação nos contextos dos cuidados primários."

- Dr. Juan Bernardo Lava, Consultor de Sistemas de Saúde Locais, USAID ReachHealth - RTI International

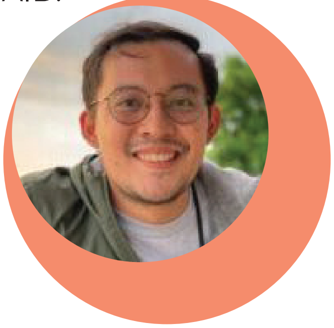

Leia o artigo original publicado na Global Health Science and Practice gratuitamente em: https://doi.org/10.9745/GHSP-D-23-00202

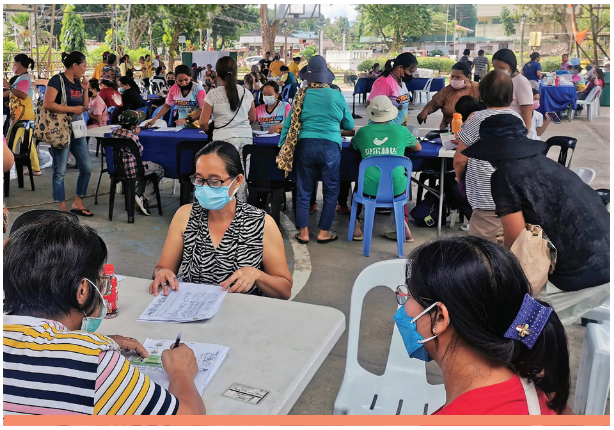

Um evento de registo no Município de San Miguel, Iloilo, que integra serviços de planeamento familiar e serviços e deteção de casos de COVID-19.

Crédito: Kaye Alfafara/USAID's ReachHealth/RTI International, 2022

Os responsáveis pela implementação e os decisores políticos podem utilizar estes resultados como um guia prático para conceber eficazmente os processos, planear os recursos, defender o apoio do governo e envolver a comunidade para integrar com êxito os serviços de saúde no sistema de cuidados primários.

Porque é que este estudo foi realizado? réalisée ? Em 2019, as Filipinas aprovaram uma lei de cobertura universal de saúde que procurava estabelecer um sistema de saúde integrado com cuidados primários como uma estratégia central da sua agenda de reforma do sistema de saúde. A pandemia de COVID-19 interrompeu os progressos iniciais no processo de reforma, mas também constituiu uma oportunidade para testar algumas intervenções e mostrar como os serviços poderiam ser integrados.

Quand et où cette étude a-t-elle été réalisée ? Este estudo realizou-se entre outubro de 2022 e abril de 2023 na província de Iloilo, nas Filipinas. A equipa de investigação tinha duas questões:

1. O facto de a vacinação contra a COVID-19 fazer parte do pacote de benefícios dos cuidados primários aumenta as taxas de vacinação?

2. A inclusão de mais serviços no pacote de benefícios leva a reembolsos mais elevados aos estabelecimentos?

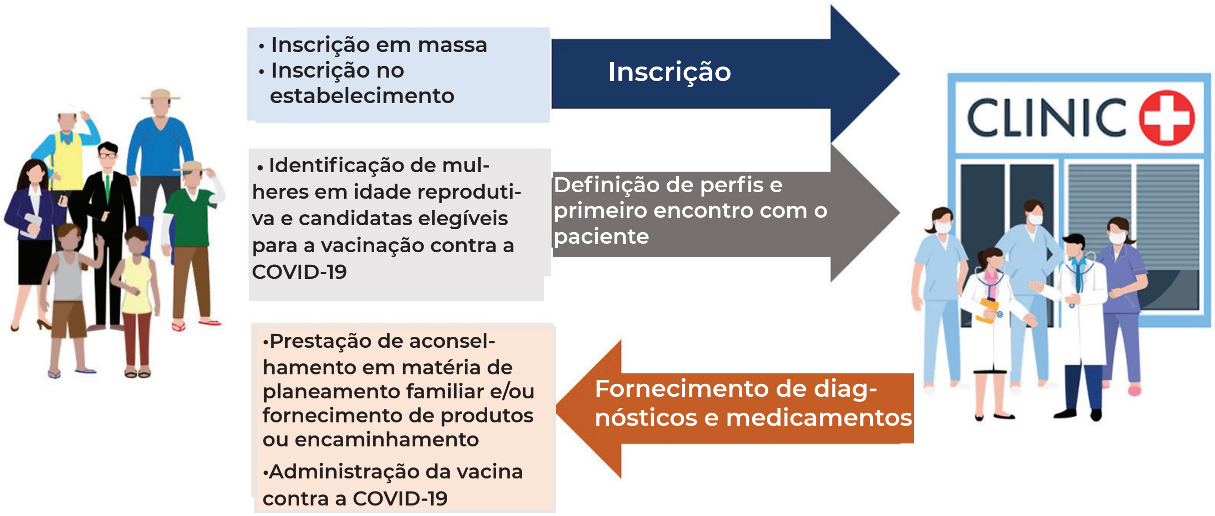

Para responder a estas questões, a equipa de investigação testou a integração da vacinação contra a COVID-19 e dos serviços de planeamento familiar no pacote de benefícios dos cuidados primários existentes em 3 pontos de entrada: quando os pacientes estavam inscritos no pacote, quando os prestadores de serviços encontravam os pacientes pela primeira vez e na fase de consulta na unidade ou encaminhamento. Em cada ponto, as mulheres em idade reprodutiva que estavam interessadas em aconselhamento e serviços de planeamento familiar e as pessoas que queriam receber a vacina contra a COVID-19 podiam receber os serviços.

USAID FROM THE AMERICAN PEOPLE

Knowledge SUCCESS

Este guia resumido é possível graças ao apoio do povo americano através da U.S. Agency for International Development ao abrigo do Acordo de Cooperação do Projeto Knowledge SUCCESS (Strengthening Use, Capacity, Collaboration, Exchange, Synthesis, and Sharing) n.º 7200AA19CA00001 com a Universidade Johns Hopkins. O Knowledge SUCCESS é apoiado pelo Gabinete de Saúde Global, da População e Saúde Reprodutiva da USAID e dirigido pelo Johns Hopkins Center for Communication Programs (CCP) em parceria com a Amref Health Africa, The Busara Center for Behavioral Economics (Busara) e a FHI 360. As informações fornecidas neste guia resumido são da exclusiva responsabilidade da Knowledge SUCCESS e não refletem necessariamente as opiniões da USAID, do Governo dos EUA ou da Universidade Johns Hopkins.
