# Supplementary material for: Integrating COVID-19 Vaccination in Primary Care Service Delivery: Insights From Implementation Research in the Philippines
Source: Glob Health Sci Pract. 2024 Feb 20;12(Suppl 1):e2300202. doi: 10.9745/GHSP-D-23-00202 (PMC10948126; doi:10.9745/GHSP-D-23-00202)
Supplement: GHSP-D-23-00202-supplement1.pdf [file GHSP-D-23-00202-supplement1.pdf]

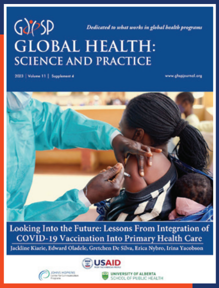

Integrating COVID-19 Vaccination in Primary Care Service Delivery: Insights From Implementation Research in the Philippines: Article Summary

Juan Bernardo Lava, Maria Socorro Quiñon, Rodney Labis, Wendell Marcelo, Miguel Angelo Lucero, Ophelia Mendoza, Laurentiu Stan, Vergil de Claro

What is this article about? COVID-19 vaccination and family planning services were integrated into the existing Philippines primary health care delivery program, which is funded by public health insurance.

What were the results? Access to health care services improved, access to family planning improved, the number of patients registered increased, and the number of first patient encounters with health care workers increased. Integrating COVID-19 vaccines into the service delivery model resulted in an additional 15,628 COVID-19 vaccinations, 46% of the target. Most of these doses were first boosters involving marginalized sections. The amount of health insurance reimbursement from PhilHealth increased from 4,000 Philippine pesos (PhP)) to PhP 553,915 within 6 months.

What do these results mean? The results offer valuable perspectives on essential factors that policymakers should consider when incorporating vaccination services into existing primary care systems. For example, using existing primary care service delivery programs and financing mechanisms offer a practical way to integrate public health interventions, such as vaccination, into primary care settings.

Implementers and policymakers can use these findings as a practical guide to effectively design processes, plan for resources, advocate for government support, and engage the community to successfully integrate health services into the primary care system.

Insights from the Authors

The ReachHealth Project is implemented by Research Triangle Institute (RTI) International in the Philippines and is funded by USAID.

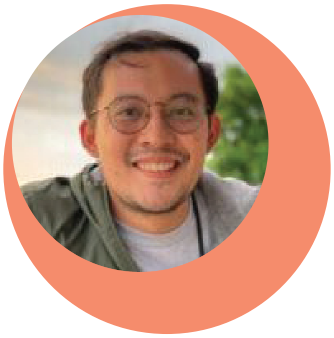

"It can be argued that the challenges arising from the pandemic have accelerated necessary and long-awaited changes in PHC systems. Therefore, it is essential to prioritize and expedite efforts to integrate vaccination services into primary care settings."

- Dr. Juan Bernardo Lava, Local Health Systems Advisor, USAID ReachHealth - RTI International

Read the original article published in Global Health Science and Practice for free at:
https://doi.org/10.9745/GHSP-D-23-00202

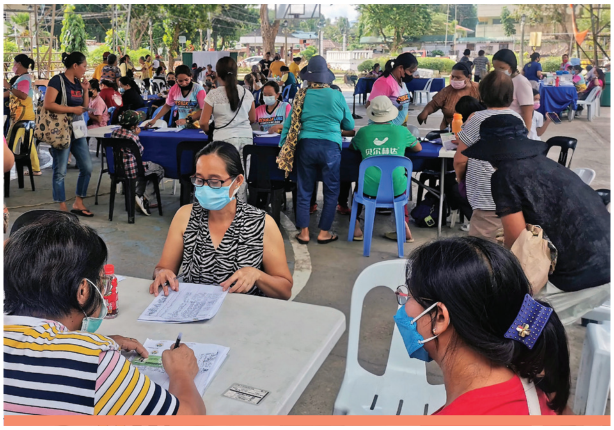

A registration event at the Municipality of San Miguel, Iloilo integrating Family Planning and COVID-19 case finding and service provision.

Credit: Kaye Alfafara/USAID's ReachHealth/RTI International, 2022

Why was this study done? In 2019, the Philippines passed a universal health coverage law that sought to establish an integrated health system with primary care as a core strategy of its health system reform agenda. The COVID-19 pandemic interrupted initial progress in the reform process, but it also presented an opportunity to test some interventions to show how services could be integrated.

When and where was this study done?
This study took place between October 2022 and April 2023 in the Province of Iloilo, Philippines.

The research team had two questions:

1. Does making COVID-19 vaccination part of the primary care benefit package increase vaccination rates?
2. Does including more services in the benefit package lead to higher reimbursements to facilities?

To answer these questions, the research team tested integrating COVID-19 vaccination and family planning services into the existing primary care benefit package at 3 entry points: when patients were registered for the package, at providers first encountered patients, and at the facility or referral stage (see figure below). At each point, women of reproductive age who were interested in family planning counseling and services and people who wanted to get the COVID-19 vaccine could receive the services.

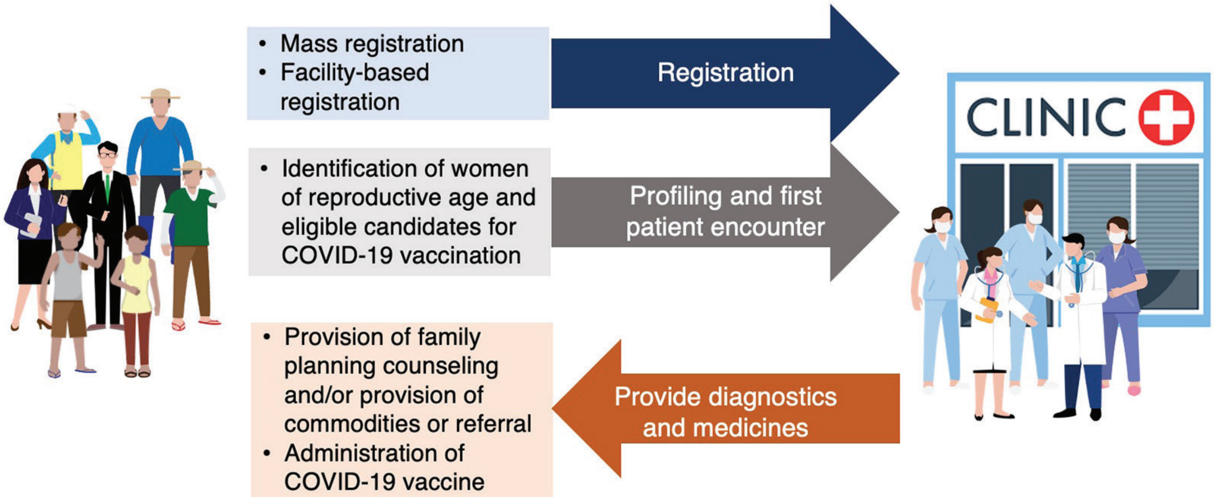

This summary brief is made possible by the support of the American People through the U.S. Agency for International Development under the Knowledge SUCCESS (Strengthening Use, Capacity, Collaboration, Exchange, Synthesis, and Sharing) Project Cooperative Agreement No. 7200AA19CA00001 with the Johns Hopkins University.
Knowledge SUCCESS is supported by USAID's Bureau for Global Health, Office of Population and Reproductive Health and led by the Johns Hopkins Center for Communication Programs (CCP) in partnership with Amref Health Africa, The Busara Center for Behavioral Economics (Busara), and FHI 360. The information provided in this summary brief are the sole responsibility of Knowledge SUCCESS and does not necessarily reflect the views of USAID, the U.S. Government, or the Johns Hopkins University.
