# Supplementary material for: Integrating COVID-19 Vaccination in Primary Care Service Delivery: Insights From Implementation Research in the Philippines
Source: Glob Health Sci Pract. 2024 Feb 20;12(Suppl 1):e2300202. doi: 10.9745/GHSP-D-23-00202 (PMC10948126; doi:10.9745/GHSP-D-23-00202)
Supplement: GHSP-D-23-00202-supplement2.pdf [file GHSP-D-23-00202-supplement2.pdf]

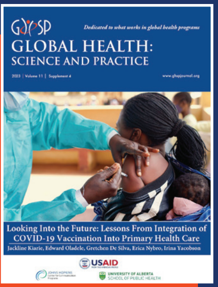

Intégration de la vaccination contre la COVID-19 dans la prestation de services de soins primaires : Perspectives de la recherche sur la mise en œuvre aux Philippines : Résumé de l'article

Juan Bernardo Lava, Maria Socorro Quiñon, Rodney Labis, Wendell Marcelo, Miguel Angelo Lucero, Ophelia Mendoza, Laurentiu Stan, Vergil de Claro

De quoi parle cet article ? Les services de vaccination et de planification familiale de la COVID-19 ont été intégrés au programme philippin de soins de santé primaires, financé par l'assurance maladie publique.

Quels étaient les résultats ? L'accès aux services de santé s'est amélioré, de même que l'accès à la planification familiale, le nombre de patients enregistrés a augmenté, ainsi que le nombre de premières rencontres entre les patients et les professionnels de la santé. L'intégration des vaccins contre la COVID-19 dans le modèle de prestation de services a permis de réaliser 15 628 vaccinations supplémentaires contre la COVID-19, soit 46 % de l'objectif. La majorité de ces doses ont constitué un premier coup de pouce pour les secteurs marginalisés. Le montant du remboursement de l'assurance maladie par PhilHealth est passé de 4 000 pesos philippins (PhP) à 553 915 PhP en l'espace de 6 mois.

Que signifient ces résultats ? Les résultats offrent des perspectives intéressantes sur les facteurs essentiels que les décideurs politiques devraient prendre en compte lorsqu'ils intègrent des services de vaccination dans les systèmes de soins primaires existants. Par exemple, l'utilisation des programmes de prestation de services de soins primaires et des mécanismes de financement existants offre un moyen pratique d'intégrer les interventions de santé publique, telles que la vaccination, dans les structures de soins primaires.

Points de vue des auteurs

Le projet ReachHealth est mis en œuvre par Research Triangle Institute (RTI) International aux Philippines et financé par l'USAID.

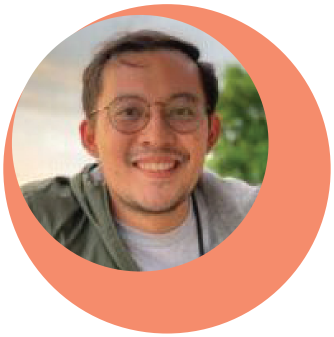

« On peut affirmer que les défis posés par la pandémie ont accéléré les changements nécessaires et attendus depuis longtemps dans les systèmes de soins de santé primaires. Il est donc essentiel de donner la priorité et d'accélérer les efforts visant à intégrer les services de vaccination dans les structures de soins primaires. »

- Dr. Juan Bernardo Lava, conseiller en systèmes de santé locaux, USAID ReachHealth - RTI International

Lisez gratuitement l'article original publié dans Global Health Science and Practice à l'adresse suivante : https://doi.org/10.9745/GHSP-D-23-00202

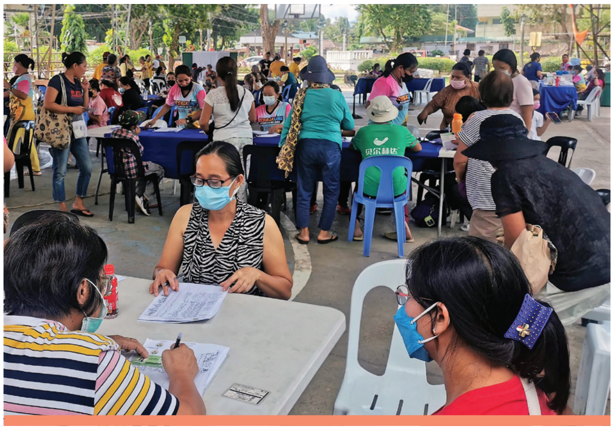

Un événement d'inscription à la municipalité de San Miguel, Iloilo, intégrant les services de planification familiale et la recherche de cas ainsi que les services liés à la COVID-19.

Crédit : Kaye Alfafara/USAID's ReachHealth/RTI International, 2022

Les responsables de la mise en œuvre et les décideurs politiques peuvent utiliser ces résultats comme un guide pratique pour concevoir efficacement les processus, planifier les ressources, plaider en faveur du soutien gouvernemental et impliquer la communauté afin d'intégrer avec succès les services de santé dans le système de soins primaires.

Pourquoi cette étude a-t-elle été réalisée ? En 2019, les Philippines ont adopté une loi sur la couverture sanitaire universelle qui vise à mettre en place un système de santé intégré avec des soins primaires comme stratégie centrale de leur programme de réforme du système de santé. La pandémie de COVID-19 a interrompu les premiers progrès du processus de réforme, mais elle a également permis d'expérimenter certaines interventions pour montrer comment les services pouvaient être intégrés.

Quand et où cette étude a-t-elle été réalisée ? Cette étude a été réalisée entre octobre 2022 et avril 2023 dans la province d'Iloilo, aux Philippines.

L'équipe de recherche était confrontée à deux questions :

1. L'intégration de la vaccination contre la COVID-19 dans l'ensemble des prestations de soins primaires permet-elle d'augmenter les taux de vaccination ?

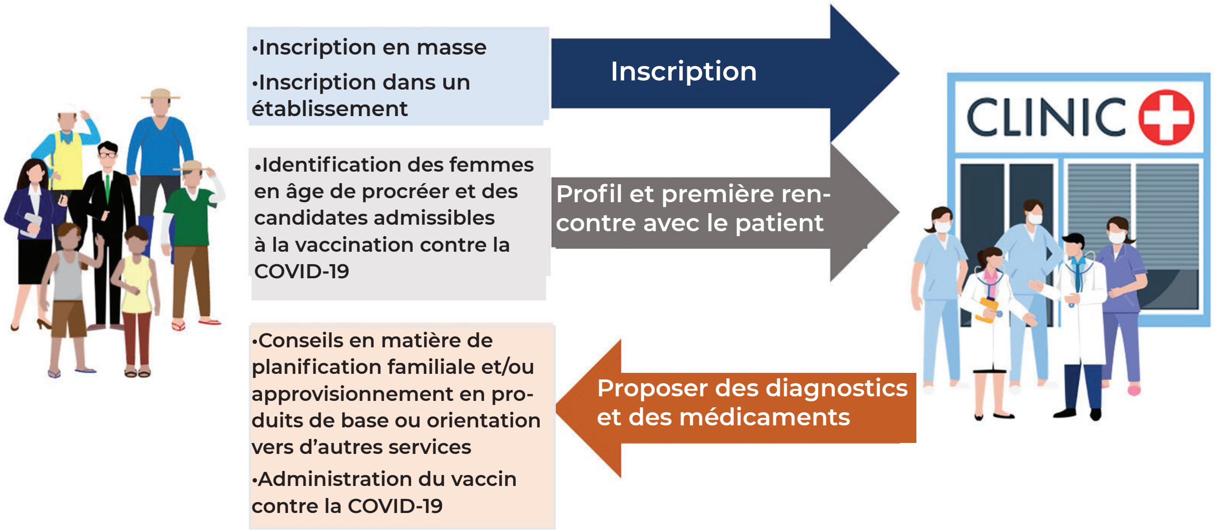

2. L’inclusion d’un plus grand nombre de services dans l’ensemble des prestations se traduit-elle par des remboursements plus élevés pour les établissements ?

Pour répondre à ces questions, l’équipe de recherche a expérimenté l’intégration des services de vaccination contre la COVID-19 et de planification familiale dans les prestations de soins primaires existantes à trois points d’entrée : lors de l’inscription des patients à ces prestations, lors de la première rencontre des prestataires avec les patients, et au niveau de l’établissement ou de l’orientation des patients. À chaque point, les femmes en âge de procréer intéressées par des conseils et des services de planification familiale et les personnes souhaitant recevoir le vaccin contre la COVID-19 pouvaient bénéficier de ces services.

USAID FROM THE AMERICAN PEOPLE

Knowledge SUCCESS

Ce guide a été réalisé grâce au soutien du peuple américain par l’intermédiaire de l’Agence américaine pour le développement international dans le cadre du projet Knowledge SUCCESS (Strengthening Use, Capacity, Collaboration, Exchange, Synthesis, and Sharing, accord de coopération n° 7200AA19CA00001 avec Johns Hopkins University. Knowledge SUCCESS est soutenu par le Bureau de la santé mondiale de l’USAID, le Bureau de la population et de la santé reproductive, et dirigé par le John Hopkins Centre for Communications Programs (CCP) en partenariat avec Amref Health Africa, le Centre d’économie comportementale de Busara et « FHI 360 ». Les informations fournies dans cette ressource relèvent de la seule responsabilité de Knowledge SUCCESS et ne reflètent pas nécessairement les opinions de l’USAID, du gouvernement américain ou de Johns Hopkins University.
